# Supplementary material for: Content-rich biological network constructed by mining PubMed abstracts
Source: BMC Bioinformatics. 2004 Oct 8;5:147. doi: 10.1186/1471-2105-5-147 (PMC528731; doi:10.1186/1471-2105-5-147)
Supplement: Additional File 2 — The original results of the above study (non-essential files are deleted to keep the file size under the limit set by BMC bioinformatics). [file 1471-2105-5-147-S2.bz2 › chilibotAdditionalFile2/dip05/358ID10779781E5741/html/IL12A_IL12B.html]

 


 **IL12A** and **IL12B** 
  
Found 14 abstracts in PubMed, retrieved 05.  
 

 What does Google say? 
 PDF only 
| .edu only 

---

**Interactive relationship** (e.g. stimulation, inhibition, etc)

**Non-interactive relationship** (e.g. studied together, co-existance, homology, etc.)

- Fetal hepatic first pass uptake of maternally derived DPHM in the paired infusion study reduces the fetal maternal plasma DPHM concentration ratio and results in significant underestimation of CLmf  [ **IL12B** ]   [ **IL12A** ] .  Ref: 9262323 J Pharmacol Exp Ther, 1997
- This study was designed to determine the reasons for this CLfm CLmf  [ **IL12B** ]   [ **IL12A** ]  difference and to identify the sites of high CLfo for DPHM.  Ref: 9262323 J Pharmacol Exp Ther, 1997
- The recently cloned cytotoxic lymphocyte maturation factor  [ **IL12A** ]  CLMF  [ **IL12B** ]   [ **IL12A** ]  also called NK cell stimulatory factor NKSF  [ **IL12A** ]  or interleukin 12 IL 12 has been described as a growth factor for mature lymphoid cells.  Ref: 7720837 Eur J Haematol, 1995
- Fetal hepatic first pass uptake may also be a factor in the underestimation of CLmf  [ **IL12B** ]   [ **IL12A** ]  for most of the other drugs.  Ref: 9262323 J Pharmacol Exp Ther, 1997
- Previous estimates of maternal and fetal placental and nonplacental clearances in pregnant sheep using a two compartment open model have revealed higher values of fetal placental clearance CLfm compared to the maternal placental clearance CLmf  [ **IL12B** ]   [ **IL12A** ]  for most drugs.  Ref: 9262323 J Pharmacol Exp Ther, 1997
- It consists of two subunits, p35 and p40, which are encoded by  **IL12A**  and  **IL12B** , respectively.  Ref: 11753820 Am J Hum Genet, 2002
- Conversely, a lower value of CLmf  [ **IL12B** ]   [ **IL12A** ]  compared with CLfm provides evidence for significant fetal hepatic uptake of these compounds.  Ref: 9262323 J Pharmacol Exp Ther, 1997
- CLmf  [ **IL12B** ]   [ **IL12A** ] , CLfm, CLmo and CLfo averaged 50.3 13. 14.4 30. 6.6 1.9 and 109.8 22.3 ml min 1 kg 1, respectively.  Ref: 9262323 J Pharmacol Exp Ther, 1997
- Interleukin 12 IL 12 previously called cytotoxic lymphocyte maturation factor  [ **IL12A** ]  CLMF  [ **IL12B** ]   [ **IL12A** ]  or NK cell stimulatory factor NKSF  [ **IL12A** ]  is a recently characterized heterodimeric cytokine composed of two subunits.  Ref: 10202188 Int J Mol Med, 1999
- Interleukin 12 IL 12 is a pleiotropic cytokine, formerly termed cytotoxic lymphocyte maturation factor  [ **IL12A** ]  CLMF  [ **IL12B** ]   [ **IL12A** ]  or natural killer cell stimulatory factor NKSF  [ **IL12A** ] , which is produced primarily by stimulated macrophages.  Ref: 7719935 Gene Ther, 1995
- When the CLmf  [ **IL12B** ]   [ **IL12A** ]  estimate is corrected for this factor and for maternal fetal DPHM plasma protein binding differences, its value approaches CLfm.  Ref: 9262323 J Pharmacol Exp Ther, 1997
